# Unravelling a pediatric enigma: coexisting retroesophageal right subclavian artery and congenital colonic stenosis masquerading as cow’s milk protein allergy and ileus in a neonate

**DOI:** 10.1186/s12887-025-05642-4

**Published:** 2025-04-03

**Authors:** Pannawat Trerattanavong, Phanthip Chaweeborisuit, Sirinat Tankruad, Aminda Hataimala, Bhannaporn Limsuksrikul, Pitchayanant Laemad, Kasidet Kittichayathon, Pakpoom Thintharua, Krai Meemon, Chinnawut Suriyonplengsaeng

**Affiliations:** 1 Department of Anatomy, Faculty of Science, Mahidol University, Bangkok, 10400 Thailand; 2 Chulabhorn International College of Medicine (CICM), Thammasat University, Pathumthani, 12120 Thailand; 3 Chakri Naruebodindra Medical Institute, Faculty of Medicine Ramathibodi Hospital, Mahidol University, Samut Prakan, 10540 Thailand

**Keywords:** Retroesophageal right subclavian artery, Congenital colonic stenosis, Gut obstruction, Cow's milk protein allergy, Ileus, Sepsis, Neonatal vomiting, Reflux, Abdominal distension, Misdiagnosis

## Abstract

**Background:**

Double alimentary tract obstruction due to congenital anomalies is a rare clinical occurrence, with limited cases published in medical literature. This article presents a unique case of coexisting retroesophageal right subclavian artery (RRSA) and congenital colonic stenosis (CCS), conditions that have not been previously documented together in pediatric population.

**Case presentation:**

A Thai male newborn was born by cesarean section at gestational age of 41 weeks. One week before birth, intrauterine asphyxia and idiopathic bilateral intracerebral hemorrhage were diagnosed by prenatal ultrasonography. Despite postnatal interventions including a ventriculoperitoneal shunt and subsequent external ventricular drain, the intracerebral hemorrhage recurred and progressed. Concurrently, the patient experienced multiple episodes of post-feeding vomiting, intermittent abdominal distension, and regular defecation without constipation. Sepsis secondary to an infected shunt occurred, accompanied by marked abdominal distension. The physician clinically suspected non-IgE-mediated cow’s milk protein allergy and ileus associated with sepsis. Tragically, the patient succumbed at seven months due to a brain abscess stemming from an infected external ventricular drain. Ultimately, postmortem examination unraveled double alimentary tract obstruction, consisting of RRSA and CCS. The RRSA, originating from proximal thoracic aorta, caused notable esophageal compression and functional stenosis which led to the frequent vomiting and reflux. The CCS involved the distal transverse colon, descending colon and proximal sigmoid colon, accounting for nearly 50% of the colon. The CCS was therefore the exact cause of intermittent abdominal distension. The stenotic colon contained submucosal and myenteric plexuses, excluding Hirschsprung disease.

**Conclusion:**

This case highlights the diagnostic complexities of RRSA and CCS resulting in double gut obstruction and masquerading as non-IgE-mediated cow’s milk protein allergy and sepsis-induced ileus. Awareness of these rare coexisting congenital anomalies can aid in early recognition, prevent misdiagnosis, enable timely management and improve outcomes for affected pediatric patients.

## Introduction

During an autopsy of a 62-year-old woman suffering from chronic dysphagia and obstructed deglutition in 1761, Dr. David Bayford discovered the aberrant right subclavian artery (ARSA) passing between the trachea and the esophagus. This significant finding led to the formal naming of the symptom “dysphagia lusoria” for the first time in medical history in 1794 [1]. The term “lusoria” is derived from the Latin “lusus naturae,” meaning “freak of nature,” reflecting the unusual nature of this vascular anomaly potentially causing esophageal compression. ARSA, also known as “arteria lusoria”, is an uncommon variant of the right subclavian artery that arises as the fourth branch of the aortic arch, distal to the left subclavian artery. It is classified into three types based on its relationship to the trachea and esophagus: pretracheal, pre-esophageal (retrotracheal), and retroesophageal types [2]. Owing to the risk of esophageal or tracheal compression by ARSA, some patients may present with symptoms such as dysphagia, emesis, cough or dyspnea. Even so, symptomatic ARSA is rarely reported in infants or children [3, 4]. The overall incidence of ARSA is 0.5–4.4% in live births [4]. Chronic vomiting in infants and children can lead to poor weight gain owing to limited oral intake, as well as an increased risk of aspiration pneumonia. Therefore, early detection is essential for effective management.

Intestinal atresia and stenosis are significant causes of neonatal intestinal obstruction. Atresia refers to a loss of lumen continuity, resulting in complete obstruction, whereas stenosis refers to a narrowing of the lumen, leading to partial obstruction. Neonates affected by these conditions may display symptoms such as constipation, abdominal distension, and/or a failure to pass meconium within 48 h after birth. Furthermore, congenital colonic stenosis (CCS) is exceedingly rare, with only 35 published cases in neonates and children since 1961 [5]. Diagnosis of CCS poses clinical challenges due to its rarity. CCS can affect any segment of the colon, though it is more commonly found in the descending and sigmoid colon, with the length of the stenotic segment ranging from 1 to 16.5 cm [5]. The symptoms of CCS vary depending on the length of the affected colon and the degree of narrowing of the colonic lumen. Delayed presentation is documented in CCS, which further complicates the diagnosis.

Instances of double alimentary tract obstruction due to congenital diseases are even rarer. These cases may involve any segment of the gastrointestinal tract, with most documented cases showing two affected sites in the lower digestive tract [6–10]. In this article, we present a case of coexisting ARSA and CCS, leading to double alimentary tract obstruction and potentially mimicking other conditions in a Thai neonate. The detailed information provided in this case report aims to raise awareness, prevent misdiagnosis and facilitate the recognition of double alimentary tract obstruction in pediatric patients.

## Case presentation

**Fig. 1:**
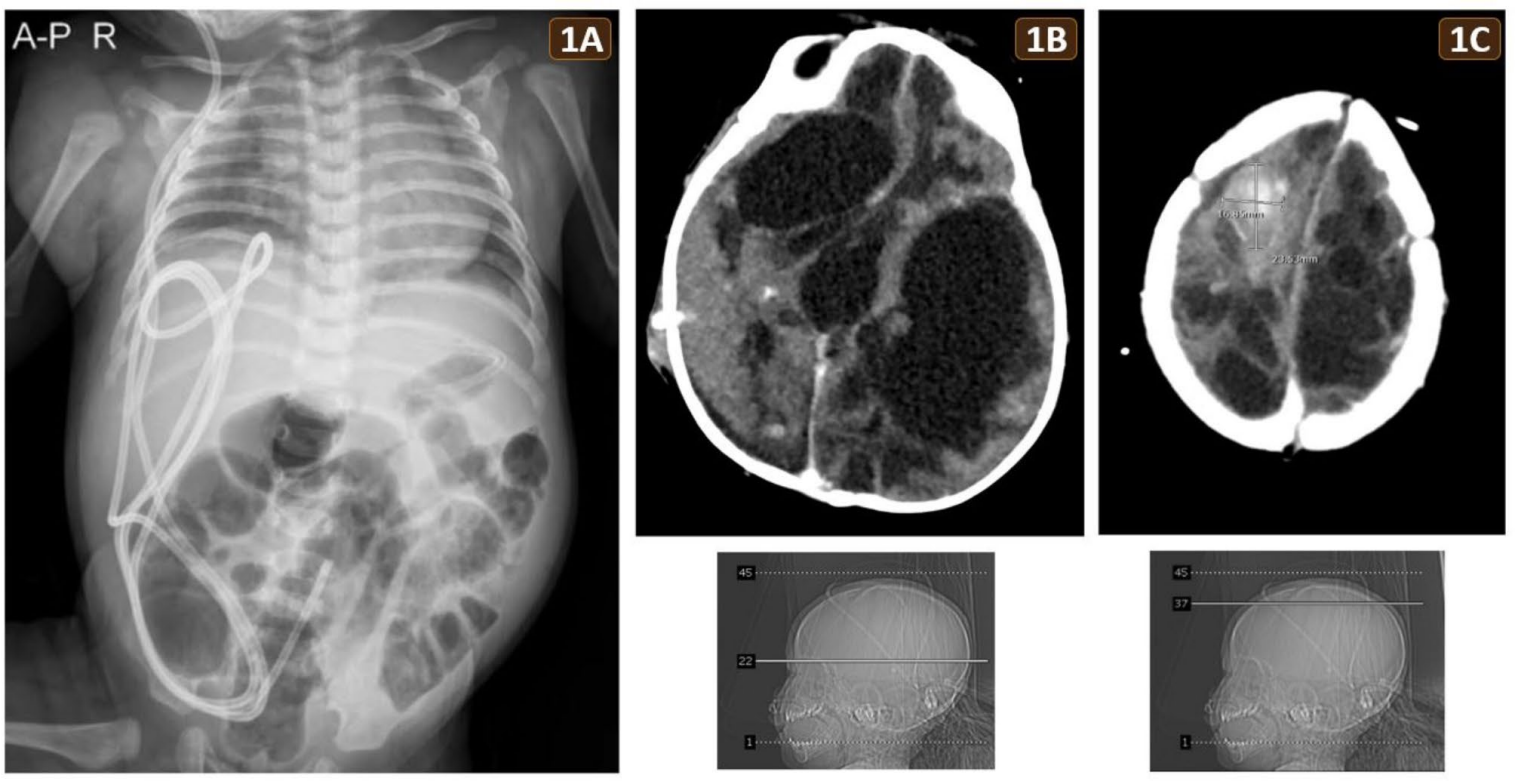
**(A)** Abdominal X-ray at 2 months of age, taken during an episode of sepsis, showing multiple dilated bowel loops without air-fluid levels or pneumoperitoneum, consistent with marked abdominal distension. The VP shunt was visible. **(B**,** C)** Brain CT at 6 months of age revealing extensive bilateral encephalomalacia, hydrocephalus, and a hyperdense lesion (2.4 × 1.7 cm) in the right high frontal lobe, indicative of recent hemorrhage

A 36-year-old Thai primigravida mother gave birth to a male newborn at a gestational age of 41 weeks by cesarean section due to maternal preeclampsia. Both parents were healthy and had no history of consanguineous marriage. The pregnancy was normal until 40 weeks of gestation when prenatal ultrasonography diagnosed bilateral intracerebral hemorrhages in the fronto-parieto-temporo-occipital regions. The newborn had a birth weight of 3,500 g. Owing to hypoxic-ischemic encephalopathy, his Apgar scores were 1, 3, and 3 at 1, 5, and 10 min, respectively, necessitating intubation and ventilatory support. A ventriculoperitoneal (VP) shunt was performed to manage obstructive hydrocephalus, yet recurrent intracerebral hemorrhage (ICH) occurred postnatally. Brain imaging after the birth did not reveal any vascular malformation, brain tumor, brain infection, or intradural venous thrombosis. Congenital coagulopathy was further excluded. After weaning off intubation and initiating oral feeding at 7 days of age, the patient experienced multiple episodes of vomiting and reflux after being fed breast milk or infant formula and exhibited intermittent abdominal distension. Stool volume ranged from 20 to 100 ml/day, and the stool consistency was runny and soft, with no history of constipation. The vomiting and reflux persisted despite switching to lactose-free and hypoallergenic infant formulas. Clinical suspicion arose for non-IgE-mediated cow’s milk protein allergy (CMPA) due to equivocal serum level of specific IgE to cow’s milk and no dermatologic symptom. At 2 months of age, the patient developed clinical signs of sepsis secondary to an infected VP shunt caused by *Staphylococcus aureus*. Concurrently, marked abdominal distension was noted. No history of necrotizing enterocolitis was documented. Abdominal X-ray radiograph showed multiple dilated bowel loops without air-fluid levels or pneumoperitoneum (Fig. 1A). Abdominal ultrasonography revealed generalized bowel dilatation without midgut volvulus or malrotation. Gut obstruction was therefore excluded. The physician suspected bowel ileus associated with sepsis and the infected VP shunt. Antibiotics were administered.

Following the removal of the infected VP shunt at the age of 3 months, right and left external ventricular drains (EVDs) were subsequently inserted. Although marked abdominal distension decreased, mild abdominal distension occurred intermittently. Additionally, few to frequent episodes of vomiting per day were consistently documented. *Serratia marcescens* ventriculitis from the infected EVD occurred at the age of 5 months. Despite the antibiotic treatment, the patient continued to suffered from brain abscesses and recurrent ICH. Brain CT at 6 months of age revealed extensive bilateral encephalomalacia, hydrocephalus and a recent hemorrhage in the right high frontal lobe (Fig. 1B and C). Both parents, acknowledging the poor prognosis, agreed to forgo resuscitation for the patient. The infant passed away at the age of 7 months, cradled in the arms of the mother. Both parents consented to donate the infant’s body as a cadaver to the Department of Anatomy, Faculty of Science, Mahidol University. Formal informed consent for cadaver preservation and postmortem examination was obtained. The infant’s body was preserved using formalin injection and embalming technique.

### Postmortem examination

**Fig. 2:**
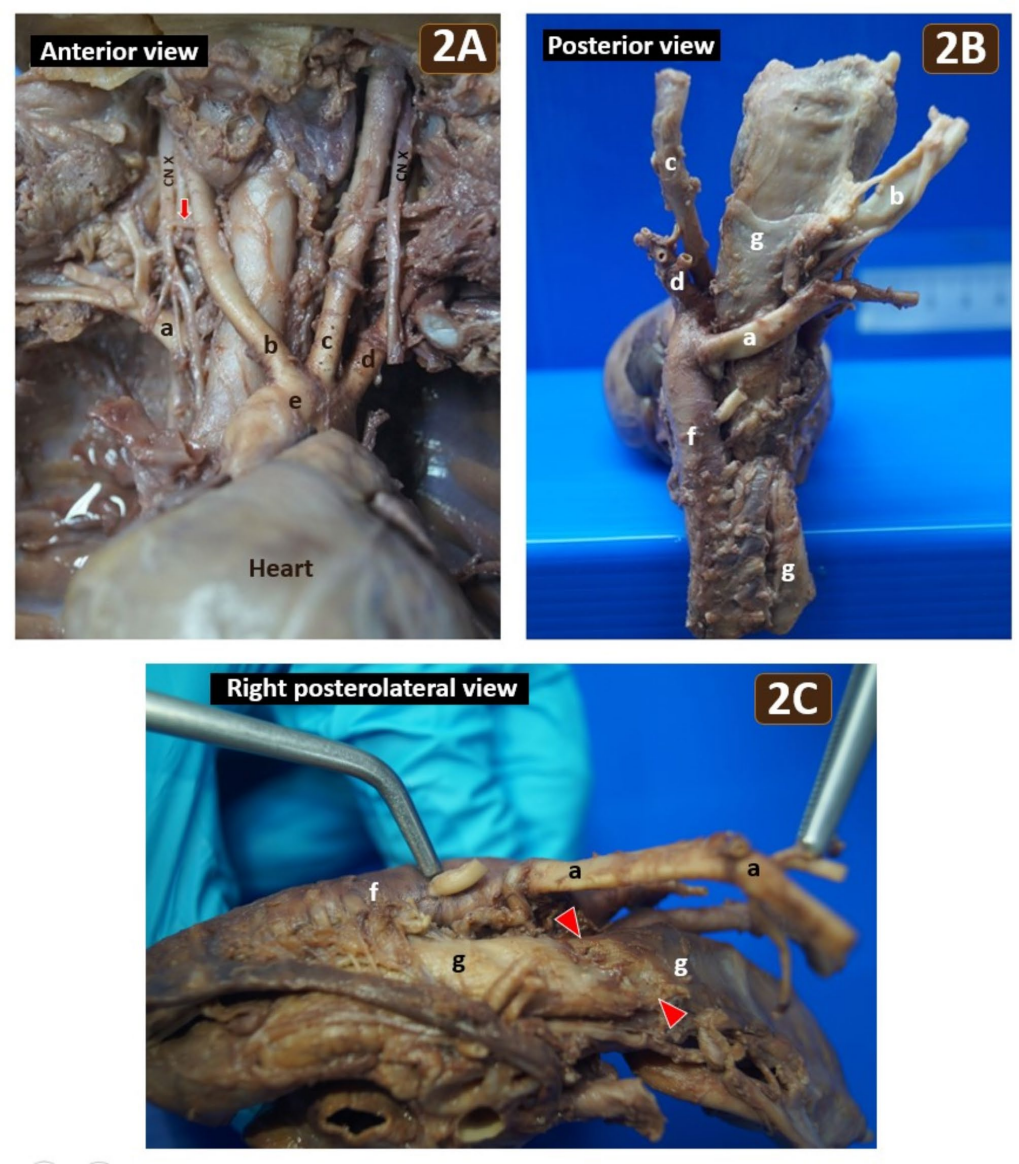
**(A)** Anterior view of the mediastinum and neck unveiled the retroesophageal right subclavian artery (a), right common carotid artery (b), left common carotid artery (c), left subclavian artery (d) and the right non-recurrent inferior laryngeal nerve (red arrow) of the vagus nerve (CN X). Arteries (b) to (d) directly originated from the aortic arch (e). No brachiocephalic trunk was observed. **(B)** Posterior view of the same specimen showing the retroesophageal right subclavian artery (a) arising from the proximal thoracic aorta (f) and coursing obliquely behind the esophagus (g). **(C)** Right posterolateral view of the same specimen illustrating notable esophageal impression (red arrowheads) caused by the retroesophageal right subclavian artery (a), which was reflected to the left

Gross examination of the thoracic and cervical regions revealed a retroesophageal right subclavian artery (RRSA) originating from the proximal thoracic aorta instead of the brachiocephalic trunk, and causing a notable compression on the posterior esophageal wall (Fig. 2). The RRSA measured 0.6 cm in diameter at its origin and 4.4 cm in length. No Kommerell diverticulum was observed. Intriguingly, the left bronchial artery originated 0.6 cm from the origin of RRSA, rather than from the thoracic aorta. The esophagus measured 1.5 cm in flat diameter, and the RRSA-to-esophagus diameter ratio was 0.4. The right inferior laryngeal branch of the right vagus nerve did not form a loop with the RRSA, and was therefore identified as the right non-recurrent inferior laryngeal nerve (Fig. 2A). The left recurrent laryngeal nerve looped around the aortic arch. No cardiac anomaly was observed.

**Fig. 3:**
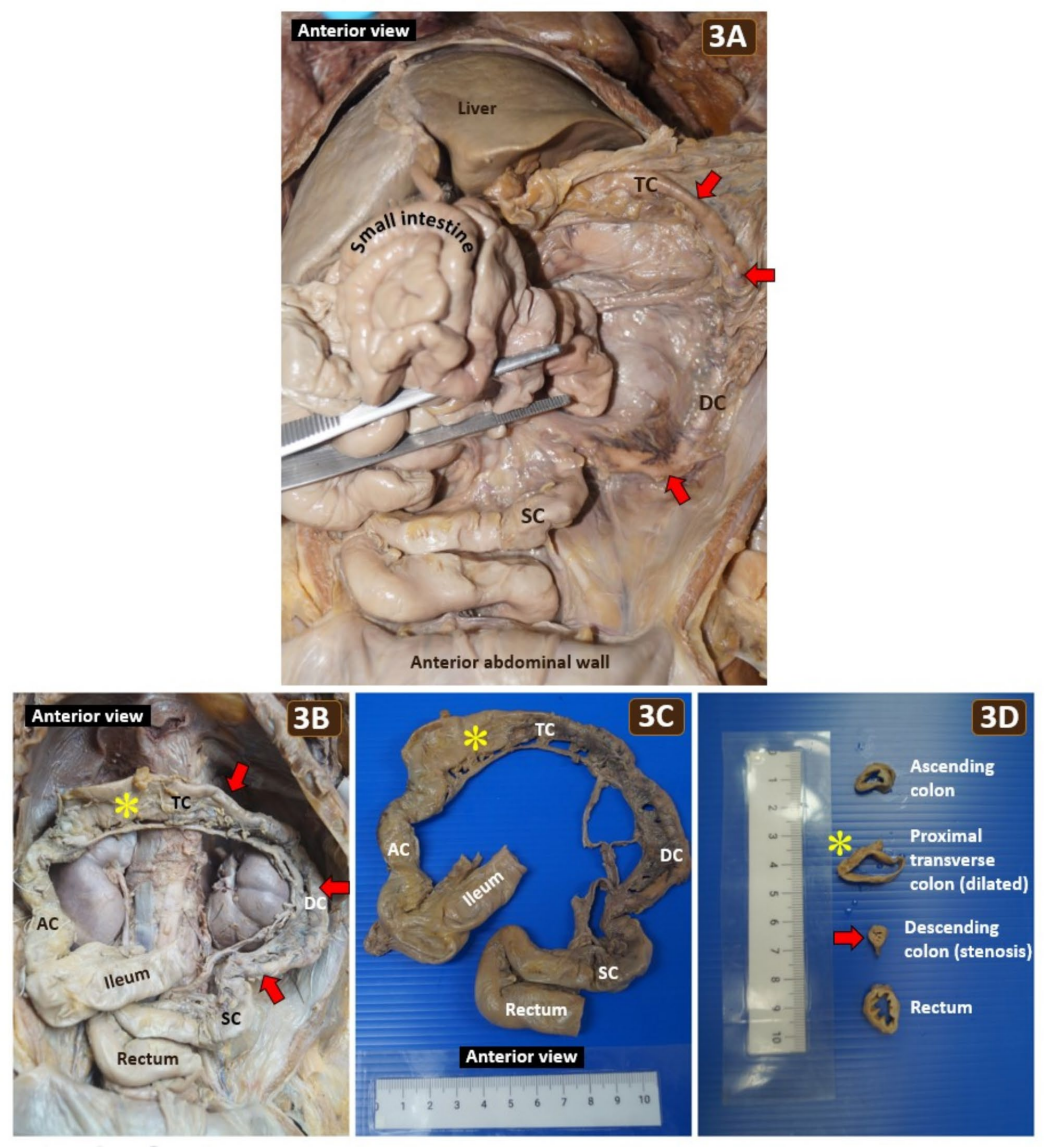
**(A)** Reflection of the small intestine revealing the in situ colonic stenosis (red arrows) affecting the distal transverse colon (TC), descending colon (DC) and proximal sigmoid colon (SC) without fibrous adhesion between abdominal viscera. **(B)** The resected distal ileum to the rectum was repositioned into the abdominal cavity to illustrate the extent of colonic stenosis (red arrows) and a dilatated segment (yellow asterisk) of the transverse colon proximal to the stenosis. **(C)** The stenotic colon, comprising nearly 50% of the total colonic length, was significantly narrower than the ascending colon (AC) and rectum. **(D)** Comparative cross sections of the ascending colon, dilated proximal transverse colon, stenotic descending colon, and rectum. Mucosal folding was absent in the dilated transverse colon (yellow asterisk). The descending colon measured 0.6 cm in diameter at the narrowest stenotic segment (red arrow), making it 40–60% narrower than the unaffected colonic segment

Gross examination of the abdomen unveiled a stenotic colon commencing from the distal half of the transverse colon to the proximal sigmoid colon measuring 15.5 cm in length (Fig. 3). The stenotic segment accounted for nearly 50% of the total length of the colon. The narrowest colonic segment measuring 0.6 cm in diameter was noted at the descending colon, while the unaffected colon ranged from 1 to 1.5 cm in diameter. The ratio of the stenotic colon’s diameter to the normal colon’s diameter ranged from 0.4 to 0.6. However, a small patent lumen was present along the stenotic colonic segment without atresia. Prior to the colonic stenosis, the proximal transverse colon showed marked dilation, measuring 2.3 cm in diameter, with loss of mucosal folding (Fig. 3D). The stomach, small intestine, mesentery, liver, pancreas, spleen, kidney, ureter and urinary bladder appeared normal. There was no pyloric stenosis, annular pancreas, intestinal atresia, gut malrotation or volvulus. No fibrous adhesion was observed between the abdominal viscera and the abdominal wall. The brain revealed bilateral encephalomalacia, ICH and dilated ventricles. Representative tissue samples from the brain, thoracic and abdominal viscera were collected.

### Microscopic examination

All tissue samples were processed, sectioned and stained with hematoxylin and eosin (H&E). Histological assessment of the esophagus, stomach, small intestine, large intestine and rectoanal canal revealed normal mucosa, submucosa, muscularis propria and serosa/adventitia.

**Fig. 4:**
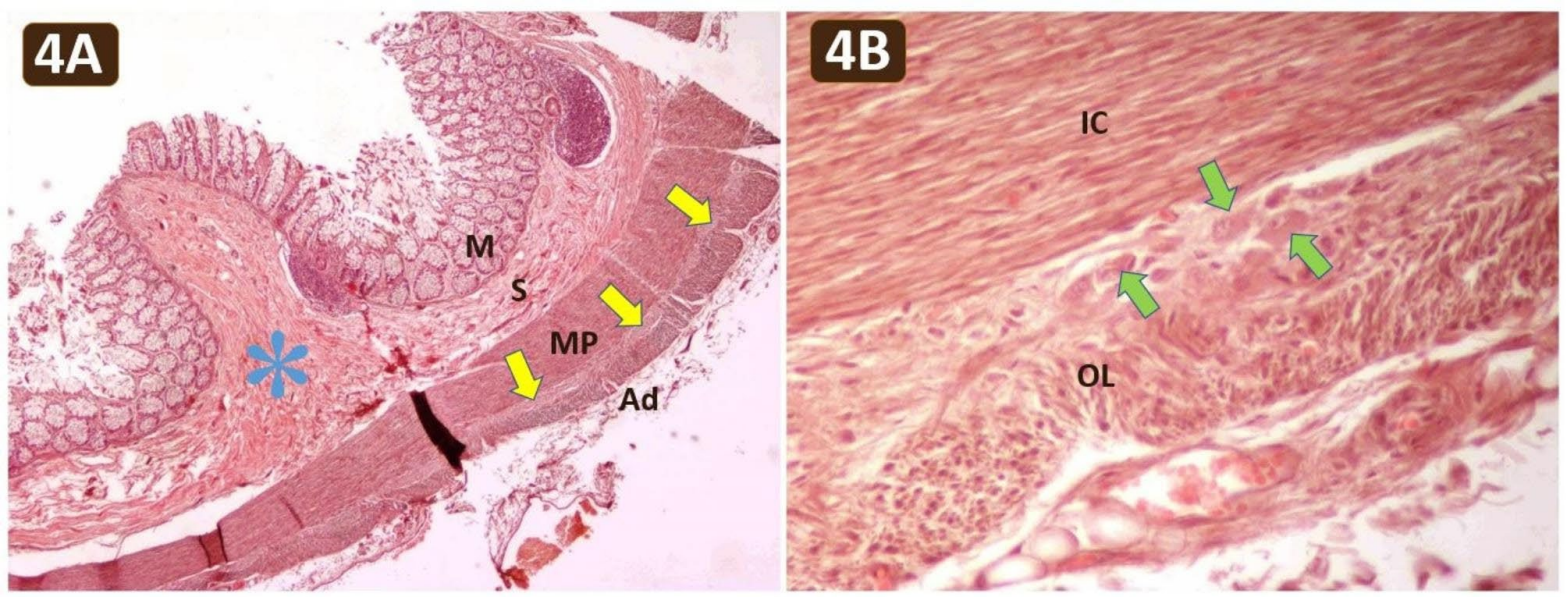
**(A)** Photomicrograph of the stenotic descending colon showing complete wall layers, including the mucosa (M), submucosa (S), muscularis propria (MP), and adventitia (Ad), along with the myenteric plexuses (yellow arrows). Submucosal fibrosis (blue asterisk) was observed in the stenotic region. H&E, 40× magnification. **(B)** A normal distribution of the ganglion cells (green arrows) in the myenteric plexuses was observed between the inner circular (IC) and outer longitudinal (OL) layers of the stenotic descending colon. H&E, 200x magnification

The stenotic colonic wall exhibited intact four layers with ganglion cells present in the submucosa and muscularis propria (Fig. 4). A submucosal fibrosis was observed in the stenotic colon (Fig. 4A). The muscularis propria remained continuous, with no segmental absence of intestinal musculature. The brain showed hematomas in both cerebral hemispheres without evidence of viral cytopathic change, vasculitis, amyloid deposition, thromboembolism or neoplasm.

## Discussion and conclusions

The mystery of frequent emesis and reflux in this case was ultimately unraveled. The postmortem examination clearly demonstrated the RRSA, a specific subtype of ARSA. Among the three types of ARSA, a retroesophageal type, as seen in this case, is the most common. This RRSA directly caused oblique esophageal compression leading to frequent vomiting after feeding, a condition known as dysphagia lusoria. In embryogenesis, ARSA arises due to the abnormal involution of the right fourth aortic arch combined with the persistence of both the right dorsal aorta and the right seventh intersegmental artery [11, 12]. ARSA is typically associated with the right non-recurrent inferior laryngeal nerve [12] which was also found in this case. The left recurrent laryngeal nerve hooked around the aortic arch, similar to normal population. Although a bulbous enlargement at the origin of ARSA, known as Kommerell diverticulum, is observed in up to 60% of ARSA cases [13], this particular case presented no Kommerell diverticulum. According to the new classification of ARSA proposed by Nedelcu AH et al. [4], ARSA in our case is classified as type Ia, with non-B and non-K. In this classification, ‘I’ refers to retroesophageal subtype, ‘a’ refers to a vertebral artery originating from ARSA, ‘B’ refers to a bicarotid trunk, and ‘K’ refers to Kommerell diverticulum. ARSA is more prevalent in some chromosomal abnormalities, notably trisomy 21, with an incidence ranging from 19 to 36% [14]. In a study by Cai M et al. [15], pathogenic copy number variations were detected in 7% of the studied cases of ARSA, especially non-isolated ARSA, with three cases showing abnormal karyotypes including Down syndrome and Edwards syndrome. However, there were no typical phenotypes of Down syndrome or Edwards syndrome in this case.

Symptomatic ARSA in the neonates and infants is rarely encountered. Only 3 out of 51 reported cases of ARSA from 2022 to 2023 involved symptomatic infants aged 1–2 years [4]. Our article highlights one of the rare cases of neonatal onset of RRSA with presenting symptoms akin to non-IgE-mediated CMPA. Although vomiting can be indicative of both non-IgE-mediated CMPA and RRSA, the former is far more prevalent in neonates compared to the latter. Consequently, physicians may not initially consider RRSA as a potential cause for vomiting and reflux in neonates. However, despite attempting multiple infant formulas to alleviate symptoms, the patient continued to experience reflux and vomiting. This persistent symptomatology should raise a red flag for physicians to consider alternative causes of vomiting. Early identification of the cause of vomiting in neonates and infants is very crucial, as it can lead to complications including inadequate weight gain due to limited oral intake and aspiration pneumonia. Imaging techniques assisting a diagnosis of ARSA include barium swallow, manometry and esophagogastroduodenoscopy. Currently, there are three major surgical procedures for repairing ARSA: open repair, thoracic endovascular aortic repair, and hybrid repair [3].

Abdominal distension, constipation and/or a failure to pass meconium within 48 h after birth are recognized as indicative symptoms of lower gut obstruction. Various diseases contributing to lower gut obstruction include intestinal stenosis or atresia, colonic pseudo-obstruction, Hirschsprung’s disease, congenital anorectal malformation, small left colon syndrome, inflammation, environmental factors or overproduction and deposition of collagen, as seen in condition like Loeys-Dietz syndrome [5, 16–20]. Colonic pseudo-obstruction is characterized by dilatation of the colon in the absence of anatomical obstruction. However, the postmortem examination in this case revealed actual colonic stenosis extending from the distal transverse colon to the proximal sigmoid colon, thus excluding colonic pseudo-obstruction. Dilatation and loss of mucosal folding of the transverse colon preceding to the colonic stenosis further confirmed true anatomical obstruction. Additionally, colonic stricture secondary to necrotizing enterocolitis was excluded due to the lack of a history of this condition. Histological examination confirmed the presence of intact submucosal and myenteric plexuses in the stenotic colon, effectively eliminating Hirschsprung’s disease from consideration. Moreover, the intact and continuous muscularis propria in the stenotic colon excludes segmental absence of intestinal musculature. Small left colon syndrome typically manifests as a transient diminutive left colon, predominantly affecting the descending and sigmoid colon, often observed in neonates born to diabetic mothers. The pathophysiology of small left colon syndrome involves neonatal hypoglycemia-induced release of glucagon, resulting in smooth muscle constriction within the left colon [16]. However, in this particular case, the affected colon extended beyond the typical regions of small left colon syndrome, reaching the distal transverse colon. Furthermore, there was no maternal history of diabetes mellitus, and the neonate presented with persistent intermittent abdominal distension lasting up to 7 months until death, a feature inconsistent with the transient obstructive symptoms typically observed in small left colon syndrome. Consequently, a diagnosis of CCS was established. From a pathology standpoint, the submucosal fibrosis observed in the stenotic colon was also consistent with CCS. The etiology of CCS remains elusive, though a prevailing hypothesis proposed by Louw and Barnard in 1955 theorizes that intrauterine vascular mesenteric compromise induces ischemia to the developing gastrointestinal tract, ultimately resulting in either intestinal stenosis or atresia depending on the severity of the ischemic insult [21].

This case presents a unique scenario wherein lower gastrointestinal obstruction primarily manifested as intermittent abdominal distension. Moreover, the daily defecation obscured the exact pathology. Despite a reduction in the stenotic colon’s diameter to 40–60% of the normal colon’s diameter, with approximately 50% involvement of CCS, the patient continued to defecate daily due to the runny and soft stool resulting from digested breast milk and infant formula. The intermittent nature of the abdominal distension in this case was contingent upon the equilibrium between the volume of daily feeding and the rate of fecal flow through the narrowed lumen of the CCS. This hypothesis elucidates why signs of lower gut obstruction were not distinctly apparent and why the possibility of lower gut obstruction had been overlooked by the physicians. In this case, the presence of CCS likely contributed to a baseline functional limitation. This limitation became more pronounced around 2 months old due to ileus, a condition that causes slowed bowel movement, which was secondary to the infected VP shunt and sepsis. This mechanism was further confirmed by the fact that the abdominal distension persisted intermittently even after the sepsis subsided. Primary anastomosis is recommended for treating CCS due to minimal variation in diameter between the proximal and distal colonic segments. However, in certain circumstances, a second procedure may be necessary [22].

Various disorders cause ICH including arteriovenous malformation, aneurysmal rupture, tumor, amyloid angiopathy, vasculitis, coagulopathy, infection, and trauma. With extensive clinical investigations and postmortem examination were conducted and the aforementioned causes were eliminated, a diagnosis of idiopathic ICH was finally rendered. An attempt to explore any potential etiological link between prenatal ICH, ARSA, and CCS was made, but no connection between these conditions was established.

This article provides a comprehensive exploration of the coexistence of RRSA and CCS, highlighting the challenges in diagnosis and management. Complete gut obstruction typically presents with more severe and easily recognizable symptoms, whereas partial gut obstruction often exhibits a wider range of symptoms that vary in severity depending on the degree of narrowing in the affected digestive tract. In this particular case, both RRSA and CCS contributed to partial obstruction of the esophagus and colon, respectively. The rarity of both conditions, especially when occurring together, makes them the least considered candidates for routine recognition. Furthermore, the complications of idiopathic ICH and sepsis added complexity to the diagnostic process. Table 1 succinctly summarizes published cases of double alimentary tract obstruction resulting from congenital gastrointestinal pathology [6–10], arranged according to the proximity of the affected gastrointestinal segments. The majority of cases involved two sites within the lower gastrointestinal tract, notably the small and large intestines, contrasting with the concurrent upper and lower alimentary tract obstruction observed in our case. Multiple sites of intestinal atresia appear to be a relatively more common cause of double alimentary tract obstruction [8, 9], with one reported case of colonic atresia alongside Hirschsprung disease [10]. Ladan et al. reported a case in which colonic atresia of the splenic flexure was diagnosed in a 3-day-old neonate, followed by the confirmation of Hirschsprung disease on day 7 of age, three days after the initial surgical removal of the atresia [10]. This underscores the challenge of diagnosing double gut obstruction when arising from distinct underlying diseases, as each may not be diagnosed simultaneously. Double CCS has also been reported [7]. Another intriguing case of double gut obstruction involves a teenage-onset presentation with double-site intussusceptions due to hamartomatous polyps in Peutz-Jeghers syndrome [6]. Notably, prenatal ultrasonography successfully diagnosed one case of double small intestinal atresia at gestational age 31 weeks, leading to immediate postnatal surgery [8]. Both sexes were affected without discernible sex predilection. Symptoms typically manifested shortly after birth. All seven cases presented symptoms of gut obstruction, including feeding difficulty, dysphagia, emesis, abdominal distension, acute abdominal pain, constipation or obstipation. While two cases ended tragically with fatality due to complications, five patients were safely discharged following surgery, underscoring the importance of prompt and proper treatment.

In conclusion, this case report unveiled a truly remarkable and unusual occurrence. While RRSA, originating from the proximal thoracic aorta, led to esophageal compression and subsequent vomiting and reflux, CCS was responsible for intermittent abdominal distension. To the best of our knowledge, the coexistence of RRSA and CCS in this Thai male newborn represented the first documented case of double upper and lower alimentary tract obstruction in a neonate. They further masqueraded as non-IgE-mediated cow’s milk protein allergy and sepsis-induced ileus. Early diagnosis is paramount because many conditions causing gut obstruction necessitate surgical intervention. Delayed diagnosis in pediatric patient leads not only to poor developmental growth, but also to more serious complications such as bowel gangrene and death. Thorough clinical evaluation with comprehensive diagnostic tests or even postmortem examination are essential to unravel the complex cases. Absolutely, even though double gut obstruction is exceedingly rare, clinicians and healthcare professionals should remain vigilant and consider it as a potential differential diagnosis, especially in neonates or infants exhibiting the aforementioned symptoms and signs. This case is presented to highlight the challenges faced and the lessons learned, assisting clinicians in recognizing similar presentations. Awareness of these rare coexisting congenital anomalies can aid in early recognition, prevent misdiagnosis, enable timely management and improve outcomes for affected pediatric patients.

**Table 1:** Summary of rare cases of double alimentary tract obstruction due to congenital anomalies

Study	Authors’ study	Davidson, et al. (2015) [6]	Shah, et al. (2019) [7]	Naidu, et al. (2021) [8]	Chen, et al. (2014) [9]	Chen, et al. (2014) [9]	Ladan, et al. (2023) [10]
**Age at onset**	Since birth (born at GA 41 weeks)	15 years old	10 days old	GA 31 weeks (born at GA 35 weeks)	10 days old	Since birth (one of preterm twins born at GA 32 weeks)	Since birth (born at GA 38 weeks)
**Sex**	Male	Female	Female	Male	Female	Female	Female
**Race**	Thai	English	Indian	Malaysian	Taiwanese	Taiwanese	Middle-eastern
**Symptom and sign**	Emesis/reflux (dysphagia lusoria), intermittent abdominal distension, no constipation	Acute abdominal pain, emesis, abdominal distension, obstipation, pigmentation on the buccal mucosa	Abdominal distension, constipation	Dilated stomach and bowel with polyhydramnios on prenatal ultrasonography at GA 31 weeks	Abdominal distension, emesis	Abdominal distension, constipation	Failure of passing meconium within the first 48 h, abdominal distension, constipation, bilious emesis
**Diagnosis**	1. Retroesophageal right subclavian artery 2. Congenital colonic stenosis 3. Idiopathic intracerebral hemorrhage with obstructive hydrocephalus	1. Double-site intussusceptions due to two hamartomatous polyps 2. Peutz-Jeghers syndrome (STK11/LKB1 mutation)	Double congenital colonic stenosis	Double small intestinal atresia	Double small and large intestinal atresia	Double colonic atresia	1. Colonic atresia 2. Hirschsprung disease
**Age at diagnosis**	1 & 2 After death 3. GA 40 weeks (by prenatal ultrasonography)	1. 5 days after the onset 2. In the same admission	1 month old	GA 31 weeks	2 months old	2 months old	1. 3 days old 2. 7 days old ( 3 days after the first surgery)
**Sites of double alimentary tract obstruction**	1. Esophagus 2. Distal transverse colon to proximal sigmoid colon	1.1 Mid jejunum 1.2 Distal ileum	1. Descending-sigmoid junction 2. Sigmoid colon	1. Second part of duodenum 2. Duodenojejunal junction	1. Terminal ileum 2. Splenic flexure	1. Hepatic flexure 2. Splenic flexure	1. Splenic flexure 2. Distal colon
**Treatment**	Blood transfusion, ventriculoperitoneal shunt and external ventricular drain (EVD)	Two small intestinal resections with primary anastomosis	Intestinal resection, end-to-end anastomosis, colostomy	Side-to-side duodenoduodenostomy, duodenojejunostomy	Enterocolostomy, colonic anastomosis	Two-stage surgery: 1. Colostomy at 4 months old 2. Colectomy with end-to-end anastomosis at 6 months old	1. Colectomy with end-to-end anastomosis; at 4 days old 2. Soave pull-through surgery; at 41 days old
**Outcome**	Died at 7 months old due to brain abscess from an infected EVD	Discharged in good condition	Discharged in good condition	Discharged in good condition	Discharged in good condition	Discharged in good condition	Died at 45 days old due to seizure and sepsis

GA: Gestational age

## Data Availability

The data and materials pertaining to this study are available upon reasonable request.

## Abbreviations

ARSA: Aberrant right subclavian artery
CCS: Congenital colonic stenosis
CMPA: Cow’s milk protein allergy
EVD: External ventricular drains
ICH: Intracerebral hemorrhage
RRSA: Retroesophageal right subclavian artery
VP: Ventriculoperitoneal

## References

[1] Bayford D. An account of a singular case of obstructed deglutition. Mem Med Soc Lond. 1794;2:275–86.

[2] Still GG, Li S, Wilson M, Wong L, Sammut P. Retrotracheal aberrant right subclavian artery: congenital anomaly or postsurgical complication? Glob Pediatr Health. 2018;5. 10.1177/2333794x18762689. 10.1177/2333794X18762689 PMC5846904 29552601

[3] Schweighofer N, Dolinšek J, Rupreht M. Arteria lusoria as a cause of dysphagia in an infant. J Pediatr Health Care. 2023;37(6):702–5. 10.1016/j.pedhc.2023.07.001. 37516943 10.1016/j.pedhc.2023.07.001

[4] Nedelcu AH, Lupu A, Moraru MC, Tarniceriu CC, Stan CI, Partene Vicoleanu SA, et al. Morphological aspects of the aberrant right subclavian artery-A systematic review of the literature. J Pers Med. 2024;14(4):335. 10.3390/jpm14040335. 38672962 10.3390/jpm14040335 PMC11051064

[5] Gupta A, Singh AK, Sunil K, Pandey A, Rawat JD, Kureel SN. Congenital colonic stenosis: A rare Gastrointestinal malformation in children. J Indian Assoc Pediatr Surg. 2021;26(5):317–23. 10.4103/jiaps.jiaps_180_20. 34728917 10.4103/jiaps.JIAPS_180_20 PMC8515529

[6] Davidson J, Wright NJ, Kufeji D. Differential diagnosis of double site intussusception in childhood: a 15-year-old Girl presenting with bowel obstruction. BMJ Case Rep. 2015. 10.1136/bcr-2015-212337. 26581705 10.1136/bcr-2015-212337 PMC4654165

[7] Shah RS, Soundharya S, Parelkar SV, Sanghvi BV, Gupta RK, Mudkhedkar KP, et al. Multiple congenital colonic stenosis - a case report and review of literature. J Pediatr Surg Case Rep. 2019;50:101279. 10.1016/j.epsc.2019.101279.

[8] Naidu RR, Hassan MR, Ruzaimie Wan Mohamad Noor WM, Md Nor MT. Double trouble with triple bubble: A rare case of double small bowel Atresia in a neonate. J Pediatr Surg Case Rep. 2021;64:101745. 10.1016/j.epsc.2020.101745.

[9] Chen H, Jiang H, Kan A, Huang L, Zhong Z, Zhang Z, et al. Intestinal obstruction due to dual Gastrointestinal Atresia in infants: diagnosis and management of 3 cases. BMC Gastroenterol. 2014;14(1). 10.1186/1471-230x-14-108. 10.1186/1471-230X-14-108 PMC4064280 24928109

[10] Ladan A, Mahdian Jouybari R, Zareh Akbari M, Moharrami Yeganeh P. Colonic Atresia and hirschsprung disease: A case report and review of the literature. J Med Case Rep. 2023;17(1). 10.1186/s13256-023-03969-z. 10.1186/s13256-023-03969-z PMC10246402 37280703

[11] Annetta R, Nisbet D, O’Mahony E, Palma-Dias R. Aberrant right subclavian artery: embryology, prenatal diagnosis and clinical significance. Ultrasound. 2022;30(4):284–91. 10.1177/1742271X211057219. 36969537 10.1177/1742271X211057219 PMC10034652

[12] Suriyonplengsaeng C, Meemon K. Retro-oesophageal right subclavian artery associated with a non-recurrent laryngeal nerve - case report. Eur J Anat. 2014;18(1):38–41. https://www.eurjanat.com/v1/journal/paper.php?id=130080km.

[13] Yang C, Shu C, Li M, Li Q, Kopp R. Aberrant subclavian artery pathologies and Kommerell’s diverticulum: a review and analysis of published endovascular/hybrid treatment options. J Endovasc Ther. 2012;19(3):373–82. 10.1583/11-3673MR.1. 22788890 10.1583/11-3673MR.1

[14] Chaoui R, Heling KS, Sarioglu N, Schwabe M, Dankof A, Bollmann R. Aberrant right subclavian artery as a new cardiac sign in second- and third-trimester fetuses with down syndrome. Am J Obstet Gynecol. 2005;192(1):257–63. 10.1016/j.ajog.2004.06.080. 15672034 10.1016/j.ajog.2004.06.080

[15] Cai M, Lin N, Fan X, Chen X, Xu S, Fu X, et al. Fetal aberrant right subclavian artery: associated anomalies, genetic etiology, and postnatal outcomes in a retrospective cohort study. Front Pediatr. 2022;10:895562. 10.3389/fped.2022.895562. 35722491 10.3389/fped.2022.895562 PMC9203729

[16] Arca MJ, Oldham KT. Atresia, stenosis, and other obstructions of the colon. In: Coran AG, editor. Pediatric surgery. Volume 2, 7th ed. Philadelphia: Elsevier; 2012. pp. 1247–53. 10.1016/B978-0-323-07255-7.00099-4.

[17] Ludwig K, Bartolo DD, Salerno A, Ingravallo G, Cazzato G, Giacometti C, et al. Congenital anomalies of the tubular Gastrointestinal tract. Pathologica. 2022;114(1):40–54. 10.32074/1591-951x-553. 35212315 10.32074/1591-951X-553 PMC9040549

[18] Maloney N, Vargas HD. Acute intestinal pseudo-obstruction (Ogilvie’s syndrome). Clin Colon Rectal Surg. 2005;18(2):96–101. 10.1055/s-2005-870890. 20011348 10.1055/s-2005-870890 PMC2780141

[19] Xie X, Xiang B, Wu Y, Zhao Y, Wang Q, Jiang X. Infant progressive colonic stenosis caused by antibiotic-related *Clostridium difficile* colitis - a case report and literature review. BMC Pediatr. 2018;18(1):320. 10.1186/s12887-018-1302-9. 30301467 10.1186/s12887-018-1302-9 PMC6178272

[20] Lim IIP, Durbin J, Tomita S. Colonic stenosis in infant with connective tissue disorder. J Pediatr Surg Case Rep. 2013;1(10):340–2. 10.1016/j.epsc.2013.09.004.

[21] Louw JH, Barnard CN. Congenital intestinal Atresia; observations on its origin. Lancet. 1955;269(6899):1065–7. 10.1016/S0140-6736(55)92852-X. 13272331 10.1016/s0140-6736(55)92852-x

[22] Khanna K, Yadav DK, Nandan R, Goel P, Rao PS. Congenital colonic stenosis with absent caecum and appendix: A rare association. BMJ Case Rep. 2018. 10.1136/bcr-2018-225072. 30389731 10.1136/bcr-2018-225072 PMC6214401

